# Gonioscopy skills and techniques

**Published:** 2022-01-31

**Authors:** Winnie Nolan, Adeola Onakoya

**Affiliations:** 1 Consultant Ophthalmologist: Moorfields Eye Hospital and Clinical Associate Professor: ICEH, London School of Hygiene & Tropical Medicine, London, UK.; 2 Professor of Ophthalmology; Head Glaucoma Services, Department of Ophthalmology, Lagos University Teaching Hospital/College of Medicine, University of Lagos Lagos, Nigeria.

**All glaucoma patients must undergo a thorough gonioscopy examination as part of their ophthalmic assessment**

Gonioscopy is a technique of viewing the iridocorneal angle: the area between the iris and cornea where the trabecular meshwork is located and where aqueous humour drains out of the eye. Gonioscopy lenses are needed to view the angle, as light from this area would not otherwise reach the observer.

Gonioscopy allows the identification of structures of the anterior chamber angle and an estimation of the angle width; it is also necessary during any procedures affecting the angle, such as laser or surgery.

Anything which impedes drainage through the trabecular meshwork can cause an increase in the intraocular pressure. It is therefore critical that all potential and newly diagnosed glaucoma patients undergo a thorough gonioscopy examination as part of their ophthalmic assessment so that the mechanism of raised intraocular pressure can be established.

In this article, we will focus on a basic gonioscopy technique for the diagnosis of primary and secondary angle-closure glaucoma and for use in angle procedures.

## Structures of the iridocorneal angle

From anterior (towards the cornea) to posterior (towards the iris), the structures seen are:

1. **Schwalbe’s line.** Demarcates Desecmet’s membrane from the anterior trabeculum. It can be located by identifying the corneal wedge (Figure 1).

**Figure 1:**
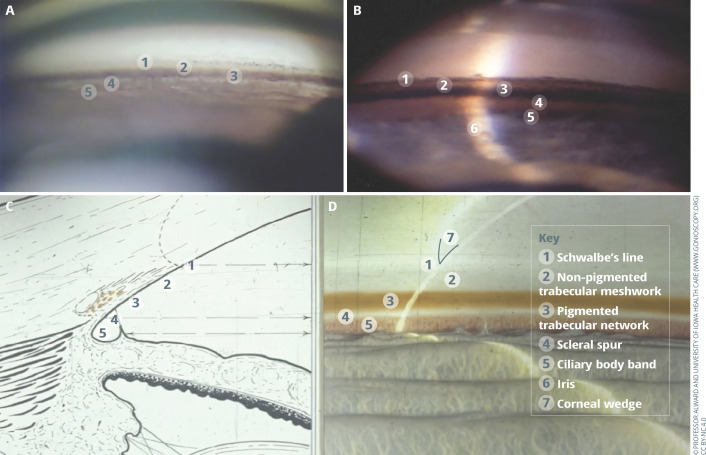
Two photographs (A and B) and two drawings (C and D) showing the structures seen on gonioscopy of an open angle. B shows a patient with pigment dispersion where the angle is densely pigmented, especially the pigmented trabecular meshwork. Some patients may have very little pigment present (hypopigmented angle) and identifying the different structures can be challenging. The bottom left image shows a cross-section of the corresponding image on the bottom right. The corneal wedge is shown where the reflections from the inner and outer aspects of the cornea meet, showing the position of Schwalbe’s line, helpful in hypopigmented angles.
2. **Non-pigmented trabecular meshwork.** A pale area adjacent to Schwalbe’s line which does not drain aqueous humour.
3. **Pigmented trabecular meshwork.** Brown/pigmented area where aqueous humour drains from the eye; it is criticial to identify whether or not it is visible on gonioscopy.
4. **Scleral spur.** A narrow, dense, whitish band posterior to the trabeculum; a consistent landmark in all eyes.
5. **Ciliary body.** A dull, brown band posterior to the scleral spur.

## Gonioscopy lenses

**Direct** gonioscopy lenses (Figure 2a), such as the Swan-Jacobs lens, act as prisms and provide a direct, panoramic view of the angle. They are used for surgical procedures on the angle, with the patient lying on their back in the operating theatre.

**Figure 2a:**
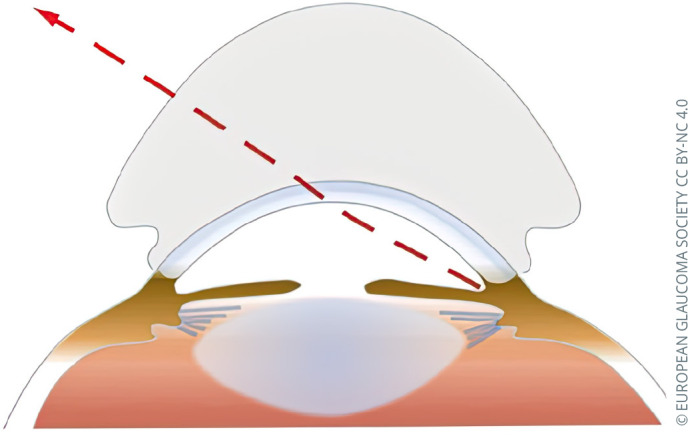
Direct gonioscopy.

**Indirect** gonioscopy lenses (Figure 2b), such as the Goldmann and Magnaview goniolenses (see the panel at the end of the article), combine a prism and a mirror to provide a reflected image of the opposite angle. Gonioscopy is carried out at the slit lamp, with the patient in a sitting position.

**Figure 2b:**
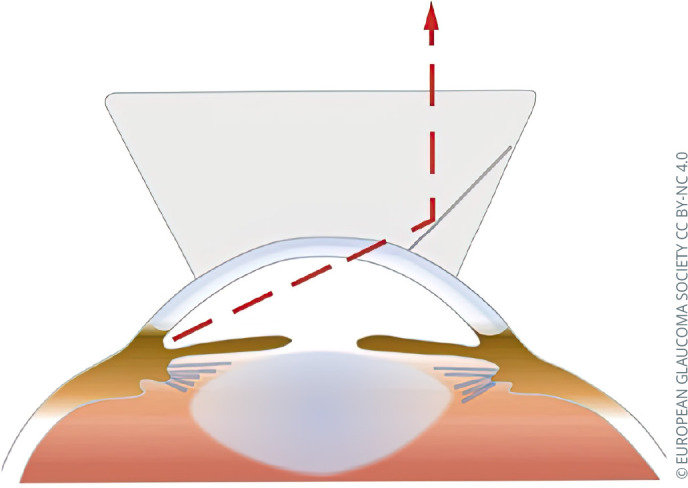
Indirect gonioscopy.

## Gonioscopy examination technique

*Some excellent videos and tutorials are freely accessible at* **www.gonioscopy.org**

**Figure 3:**
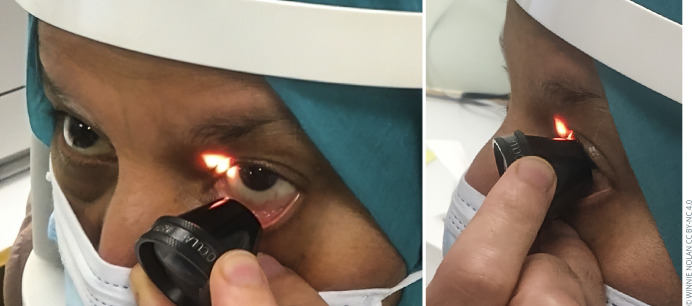
Insertion of an indirect gonioscopy lens using coupling gel.
- To make insertion easier, the forefinger of the hand inserting the lens can be used to pull the lower lid down and if necessary, the thumb of the other hand to elevate the upper lid.
- Once the lens is in, ask the patient to look straight ahead.
- View the inferior angle through the superior mirror, and vice versa.
- Rotate the lens to view the nasal and temporal angles. In order to best visualise the trabecular meshwork and other angle structures, the slit beam should be at right angles to the mirror and the light offset by 30–60 degrees.

- Ensure minimal lighting in the room and a short (1 mm) slit beam to avoid artifactual opening of the angle (bright illumination will cause pupil constriction and opening of the angle).
- Instil topical anaesthesia and explain the procedure to the patient.
- Instruct the patient to keep both eyes open as this results in less squeezing of the eye to be examined.
- For less experienced practitioners, we suggest using an indirect gonioscopy lens with coupling gel as a more stable view is gained.
- Apply a coupling gel to the lens (e.g., carbomer gel).
- Instruct the patient to look up.
- Insert the inferior rim of the lens onto the surface of the eye and then quickly apply the rest of the lens rim to the globe (Figure 3).

**“A good method of locating trabecular meshwork is to identify Schwalbe’s line (see Figure 1) and then move posteriorly.”**

## Assessing the angle width and status

First establish whether the angle is open or closed. Iridotrabecular contact (ITC) is present when it is not possible to visualise the pigmented trabecular meshwork without manipulation. A good method of locating trabecular meshwork is to identify Schwalbe’s line (see Figure 1) and then move posteriorly.

Asking the patient to move the eye in the direction of the mirror, indenting the cornea, or increasing the light can all help to open the angle and visualise more posterior structures. These manoeuvres can help to differentiate between appositional angle closure as compared to synechial closure. However, grading of the angle width (how open the angle is) should be performed in dim light, with the eye in the primary position and without indentation.

**Figure 4:**
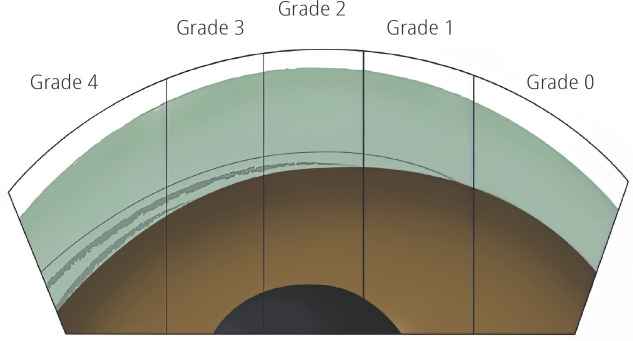
Shaffer grading system.

There are a number of different grading systems for angle assessment (see references). In practice, a modified Shaffer grading scheme (Figure 4) is commonly used for grading the angle width. The visibility of angle structures is used:

- **Grade 0.** No angle structures visible.
- **Grade 1.** Schwalbe’s line visible (i.e., the angle is essentially closed as aqueous humour is not able to drain).
- **Grade 2.** Pigmented trabeculum visible (aqueous able to drain but the angle is relatively narrow).
- **Grade 3.** Scleral spur visible.
- **Grade 4.** Ciliary body visible (angle is wide open).

**Figure 5:**
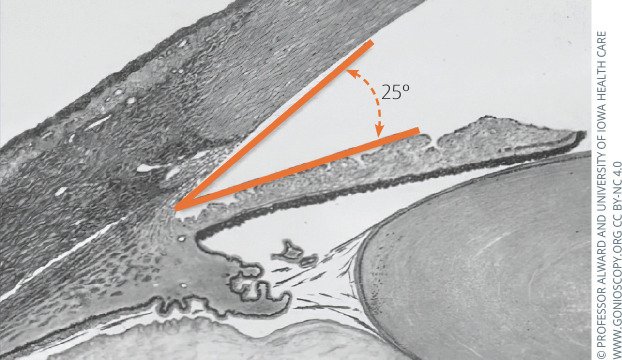
Angle width of 25 degrees.

Each quadrant (superior, inferior, nasal and temporal) should be graded. Although this is a quick and easy system it is also helpful to also estimate the angle width in degrees (Figure 5) as it provides more information on risk of future closure.

**“It is important to consider the whole clinical situation.”**

When assessing whether angle closure is present, or if the patient is at high risk of angle closure, it is important to consider the whole clinical situation (clinical history, symptoms, other examination findings, and so on). For practical purposes, if the angle width is greater than 25 degrees and scleral spur is visible all the way around the angle, there is likely to be a low risk of angle closure. If pigmented trabecular meshwork cannot be seen, i.e, there is iridotrabecular contact for more than two quadrants (over half) of the angle on gonioscopy, there is likely to be a high risk of angle closure. In this situation, intervention to open the angle may be warranted, depending on the presence of other abnormalities such as raised intraocular pressure or risk factors for acute angle closure.

**Indirect gonioscopy lenses**

**Figure 6:**
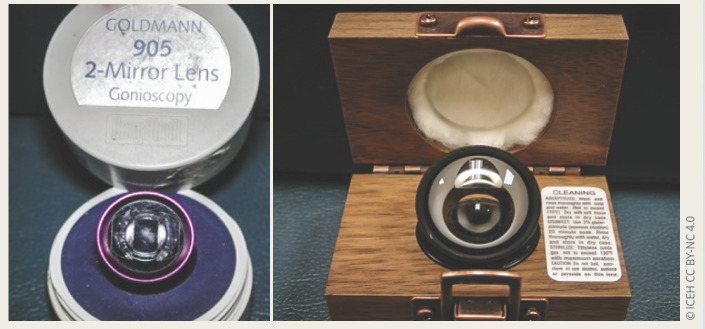
Image on the left shows a Goldmann two mirror lens, on right is a Magnaview one mirror lens

Goldmann and Magnaview lenses are indirect gonioscopy lenses, or goniolenses (Figure 6), which require the use of a coupling gel to fill the gap between the lens and the cornea and give a stable, undistorted view of the angle structures and configuration. These lenses have either one or two mirrors through which the observer views the angle. The Magnaview lens is larger than all the Goldmann lenses, giving more magnification and allowing a more detailed view of the angle. However, it may be difficult to insert if patients have a small palpebral aperture. It can be used for delivering selective laser trabeculoplasty.

The Zeiss, Posner and Sussman lenses are indirect gonioscopy lenses which allow a rapid view of the entire angle without the need for a coupling gel. They can be used for indentation gonioscopy – pressure is applied on the cornea with the lens, this can help to open up the angle so that further structures can be identified (such as with plateau iris syndrome). If peripheral anterior synechiae are present the angle will not open further even with indentation. The view of the angle is not as stable or clear as that with the Goldmann or Magnaview. Inadvertent indentation can result in corneal striations and distortion of the view as well as accidental opening up of the angle and misclassification of a closed angle as open.
